# Phenotypic and functional consequences of haploinsufficiency of genes from exocyst and retinoic acid pathway due to a recurrent microdeletion of 2p13.2

**DOI:** 10.1186/1750-1172-8-100

**Published:** 2013-07-10

**Authors:** Jiadi Wen, Fátima Lopes, Gabriela Soares, Sandra A Farrell, Cara Nelson, Ying Qiao, Sally Martell, Chansonette Badukke, Carlos Bessa, Bauke Ylstra, Suzanne Lewis, Nina Isoherranen, Patricia Maciel, Evica Rajcan-Separovic

**Affiliations:** 1 Child and Family Research Institute, Department of Pathology, University of British Columbia, Vancouver, BC, Canada; 2 Life and Health Sciences Research Institute (ICVS), School of Health Sciences, University of Minho, Braga, Portugal; 3 ICVS/3B’s - PT Government Associate Laboratory, Braga/Guimarães, Portugal; 4 Center for Medical Genetics Dr. Jacinto Magalhães, National Health Institute Dr Ricardo Jorge, Porto, Portugal; 5 Genetics, Trillium Health Partners, Credit Valley Hospital Site, Mississauga, ON, Canada; 6 Department of Pharmaceutics, School of Pharmacy, University of Washington, Seattle, WA, USA; 7 Department of Pathology, VU University Medical Center, Amsterdam, The Netherlands; 8 Child and family Research Institute, Department of Medical Genetics, University of British Columbia, Vancouver, BC, Canada

**Keywords:** 2p13 deletion, EXOC6B, CYP26B1, Developmental delay, Cranial/skeletal anomalies

## Abstract

**Background:**

Rare, recurrent genomic imbalances facilitate the association of genotype with abnormalities at the “whole body” level. However, at the cellular level, the functional consequences of recurrent genomic abnormalities and how they can be linked to the phenotype are much less investigated.

**Method and results:**

We report an example of a functional analysis of two genes from a new, overlapping microdeletion of 2p13.2 region (from 72,140,702-72,924,626). The subjects shared intellectual disability (ID), language delay, hyperactivity, facial asymmetry, ear malformations, and vertebral and/or craniofacial abnormalities. The overlapping region included two genes, *EXOC6B* and *CYP26B1*, which are involved in exocytosis/Notch signaling and retinoic acid (RA) metabolism, respectively, and are of critical importance for early morphogenesis, symmetry as well as craniofacial, skeleton and brain development. The abnormal function of *EXOC6B* was documented in patient lymphoblasts by its reduced expression and with perturbed expression of Notch signaling pathway genes *HES1* and *RBPJ*, previously noted to be the consequence of *EXOC6B* dysfunction in animal and cell line models. Similarly, the function of *CYP26B1* was affected by the deletion since the retinoic acid induced expression of this gene in patient lymphoblasts was significantly lower compared to controls (8% of controls).

**Conclusion:**

Haploinsufficiency of CYP26B1 and EXOC6B genes involved in retinoic acid and exocyst/Notch signaling pathways, respectively, has not been reported previously in humans. The developmental anomalies and phenotypic features of our subjects are in keeping with the dysfunction of these genes, considering their known role. Documenting their dysfunction at the cellular level in patient cells enhanced our understanding of biological processes which contribute to the clinical phenotype.

## Background

Chromosomal abnormalities involving the 2p13 chromosomal region were detected previously in subjects with developmental delay using traditional chromosome testing [1,2]. Abnormal features present in at least 50% of cases included abnormal head size/shape, nose, ears, chest and vertebral, digital and genital anomalies. The involvement of the *CYP26B1* gene was suggested in a subject with a *de novo* inversion involving 2p13 and 2q34, who had a Klippel-Fiel anomaly (vertebral fusion of cervical spine), psychomotor retardation, speech limitation, facial asymmetry, ear abnormalities, and scoliosis [3,4]. Disruption of *EXOC6B* and its fusion with *TNS3* from 7p12 due to a translocation t(2;7)(p13;p12), was considered to be a possible cause of the intellectual disability, ADHD and congenital abnormalities (renal malformation, microcephaly, long bone diaphyseal broadening) in a subject reported by Borsani *et al.*[5].

*CYP26B1* (cytochrome P450, family 26, subfamily B, polypeptide 1) is one of the three CYP26 gene isoforms (*CYP26B1*, *CYP26A1* and *CYP26C1*) which encode the cytochrome-P450 enzymes that catabolize retinoic acid (RA) [6]. RA is the principal active metabolite of vitamin A and is an essential component of cell-cell signaling during vertebrate organogenesis [7]. Too little or too much RA causes the human malformation syndrome associated with vitamin A deficiency (VAD) or RA embryopathy, which includes craniofacial (e.g. ears, eyes, facial asymmetry), central nervous, musculoskeletal, and urogenital abnormalities [8-12]. Homozygous knockout of *CYP26B1* in animals has been associated with postnatal mortality, abnormal craniofacial, limb and gonadal development [13,14], while a conditional deletion (controlled expression) of *CYP26B1* resulted in a less severe phenotype. The difference in the phenotypes was attributed to differing levels of activated retinoid signaling [15]. Although deletions of the whole *CYP26B1* gene have not yet been reported in humans, homozygous point mutations of *CYP26B1* were reported in two families, resulting in prenatal and early postnatal lethality, skeletal and craniofacial abnormalities, fusion of long bones, calvarial bone hypoplasia and sutural defects, resulting in craniostenosis and brachycephaly [16]. *CYP26B1* mutations in both families led to a significantly attenuated ability to metabolize exogenously applied retinoic acid, confirming the impact of dysfunction of this gene on RA metabolism. Previous studies have shown that *CYP26B1* is responsible for ATRA (all-trans retinoic acid) clearance in several tissues [17]. For example in T-cells, *CYP26B1* is the only CYP26 enzyme up-regulated by ATRA and its expression regulates retinoic acid dependent signals in T-cells [18]. In addition, in many human cell lines *CYP26B1* mRNA is inducible by retinoic acid treatment while in rodents cyp26b1 expression correlates with dietary vitamin A intake [19-22].

*EXOC6B* (exocyst complex component 6B) encodes a protein homologous to Sec15 in *Saccharomyces cervisiae*. It belongs to a multiprotein complex (exocyst) required for targeted secretion (exocytosis) which is crucial for cell polarity, growth and communication [23]. In *Drosophila*, sec15 promotes Notch signaling, through specific vesicle trafficking of delta ligand, and has a role in asymmetric division of sensory precursors and neuronal fate determination [24]. *Drosophila* neurons with sec15 mutations show loss of synaptic specificity and mislocalization of proteins known to affect synaptic specificity in photoreceptors [25]. Recently, Guichard *et al.* showed reduced transcription of Notch signaling effectors *HES1* and *RBPJ* in knockdown *Drosophila* sec15 and in a human brain microendothelial cell line with abnormal *EXOC6*B function [26]. Notch signaling plays a pivotal role in the regulation of many fundamental cellular processes, such as acquisition of specific fates in context-dependent manner, differentiation and lineage decisions during embryonic development, neurogenesis, as well as morphogenesis involving regulation of left-right asymmetry [27,28]. Perturbations of the Notch pathway have been reported in human developmental disorders which demonstrate a variety of symptoms, including ID and/or skeletal abnormalities (e.g. Allagile syndrome and spondylocostal dysostosis [29,30]).

We report the first description of the clinical and functional consequences of hemizygous deletion of the two genes, *CYP26B1* and *EXOC6B,* located in chromosome region 2p13, and which are respectively, involved in retinoic acid and Notch signaling pathways of critical importance to normal human fetal development.

## Materials and methods

### Whole genome array CGH analysis

Genomic DNA was extracted from peripheral blood using the PUREGENE DNA Isolation Kits (Gentra, Minneapolis, MN) for Subject 1 and Citogene^®^ DNA isolation kit (Citomed, Portugal) for Subject 2**.** For Subject 1, Agilent 105 K array (version 4.0, June 2006, Agilent Technologies, CA, USA) analysis was performed as previously described.^25^ CNV selection was done by Agilent DNA Analytics (version 3.5.14, Agilent Technologies) using the ADM-2 algorithm (cutoff 6.0), followed by a filter to select regions with three or more adjacent probes and a minimum average log2 ratio + 0.25 [31]. The deletion of Subject 1 was *de novo,* as determined by FISH using probe RP11-91 F23.

For Subject 2, the aCGH analysis was performed using a CGH Agilent 180 K custom array design accessible through the gene expression omnibus (GEO) accession number GPL15397. Previously published protocol was used [32]. Image analysis was performed using the across-array methodology described previously [33]. CGH data was analyzed using Nexus Copy Number 5.0 software with FASST Segmentation algorithm. The deletion was determined to be de novo using the same array for both parents.

For both cases, the array design, database consultation and comparative analysis was performed using genome build 36.1/HG18.

### Functional studies

Immortalized EBV transformed lymphoblastoid cell lines (LCL) from Subject 1 were cultured in RPMI media containing 10% FBS, 50 units/mL penicillin, 50 μg/mL streptomycin. Cells were maintained in a humidified 37°C incubator with 5% CO_2_. Control cells were obtained from healthy subjects, the majority of which aged 28–50 years.

#### RNA expression

Total RNA was extracted using an RNeasy Plus Mini kit (Qiagen) from Subject 1-derived LCLs and whole blood collected in Tempus tubes. Aliquots (~500 ng) of the total RNA extracts prepared from were subsequently reverse-transcribed into cDNA using GeneAmp Gold RNA PCR Core Kit (Applied Biosystems).

The expression of *EXOC6B* and *CYP26B1* was first assessed in RNA extracted from control whole blood and control LCLs by real-time qPCR using the ABI PRISM 7300 Sequence Detection System (Perkin-Elmer Applied Biosystems). The specific nucleotide sequences for primers of *EXOC6B* and *CYP26B1* were as follows: *EXOC6B* Forward 5’-GAC CTC ATT GCC TTT CTT CGT A-3’, Reverse 5’-CAA GCT GAC ATA CAC GCT GT-3’(mapped to exons 18–19); *CYP26B1* primer set 1; Forward 5’-ACA CAG GGC AAG GAC TAC T-3’, Reverse: 5’-GCA TAG GCC GCA AAG ATC A-3’(mapped to exon 4–5); *CYP26B1* primer set 2: Forward: 5’-CTA CCT GGA CTG CGT CAT CA-3’, Reverse: 5’-CCC GGA TGC TAT ACA TGA CA-3’ (mapped to exon 5–6). *EXOC6B* had a detectable transcript, while no transcript was detected with *CYP26B1* primers for exons 4–5 and exons 5–6 in control whole blood, nor transformed lymphoblasts.

Real-time qPCR was performed for *EXOC6B* and its downstream genes *HES1* and *RBPJ* using SYBR^®^ Green PCR Master Mix (Applied Biological Materials Inc). Primer sequences were as follows: *HES1* Forward: 5’-GAG CAC AGA AAG TCA TCA AAG C-3’ (mapped to exons 1–3), Reverse: 5’-TTC CAG AAT GTC CGC CTT C-3’; *RBPJ* Forward: 5’-GGG ATA GGA AAT AGT GAC CAA GA-3’, reverse: 5’-GTG CTT TCG CTT GTC TGA GT-3’ (mapped to exons 7–8).

Quantification of expression level of *EXOC6B, HES1* and *RBPJ* was performed in comparison to actin. All PCR reactions were performed in triplicate, with the mean of 2^-ΔΔCt^ values [34] being used to determine mRNA levels for Subject 1 in comparison to the mean expression level for three controls. Significance was calculated using the Student’s t test (VassarState Statistical Computation website). Values were considered statistically significant with a p-value of <0.05.

#### Protein expression

LCLs were washed three times in PBS and incubated in 1 mL RIPA buffer (Thermo Scientific, USA), supplemented with Halt Phosphatase Inhibitor Cocktail (Thermo Scientific, USA) for 15 minutes on ice. Cell lysates were centrifuged at 10,000 × g for 15 minutes at 4°C, and the supernatants were used for Western blotting. Cell lysate protein concentrations were determined using a DC™ Protein Assay (BioRad laboratories, USA). Western blots containing cell-lysate aliquots (~30 μg) were prepared and immunoblotted using a polyclonal antibody directed against human RBPJ or a monoclonal antibody against human HES1 (Abcam, Cambrige, MA, USA). Three antibodies against human EXOC6B were tested in this study (Sec15B(C-14) (goat polycolonal antibody):sc-34375, Santa Cruz Biotechnology,Inc. Anti-EXOC6B antibody (rabbit polycolonal antibody): ab116383, Abcam Inc. Anti-EXOC6B antibody (mouse polycolonal antibody): H00023233-B01P, Novus Biologicals) but did not provide a clear or visible band at the expected size, amenable to interpretation. To standardize the amounts of protein loaded into each lane, the blots were reprobed with a monoclonal antibody directed against human β-actin (NovusBiologicals, Littleton, USA). The ECL Western Blotting system was used to detect the amount of each antibody bound to antigen and the resultant photographic films were analyzed by UV densitometry (GE Healthcare Life Sciences, Pittsburgh, USA). The absorbance values obtained for HES1 and RBPJ were then normalized relative to the corresponding β-actin absorbance value. The average of HES1 or RBPJ protein expression was obtained from three independent replicates from the Subject and the control samples. P values for two tailed Student test were considered statistically significant if <0.05.

#### CYP26B1 expression upon ATRA induction

Cells from seven control subjects (aged 2–42 years) and Subject 1 were plated in 6-well plates at a density of 400,000 cells/mL in 3 mL and treated in triplicate with 1 μM *all-trans* retinoic acid (ATRA) or an equal volume of vehicle (ethanol) in triplicate and incubated in the dark for 72 hours. Following treatment, cells were pelleted and resuspended in 1 mL Tri-Reagent Solution (Life Technologies, Grand Island, NY) followed by repeated pipetting to lyse the cells. RNA was then isolated according to manufacturers instructions and RNA quantity and quality were determined using a NanoDrop 2000c spectrophotometer (Thermo Scientific, Waltham, MA) by measuring the absorbance at 260 nm and 280 nm. Complimentary DNA (cDNA) was generated from 1 μg total RNA by reverse transcription using the TaqMan reverse transcription reagents kit (Life Technologies, Grand Island, NY), as previously described [17,35]. TaqMan Gene Expression Master Mix, PCR primers, and fluorescent probes were obtained from Applied Biosystems (Life Technologies, Grand Island, NY). Probes were labelled with the 5’ reporter dye FAM (*CYP26A1* and *CYP26B1*) or VIC (GAPDH). Primer probe pairs used were: *CYP26A1* (Hs00219866_m1), *CYP26B1* (Hs00175627_m1), and the endogenous control *GAPDH* (RefSeq: NM_002046.3). Quantitative real-time PCR was conducted on a StepOnePlus Real-Time PCR instrument (Applied Biosystems, Foster City, CA) as previously described [17,35]. The fold-increase in *CYP26A1* and *CYP26B1* mRNA was calculated using the ΔΔC_t_ method (fold difference = 2^-ΔΔCt^) by comparing the ATRA-treated cells to the vehicle treated controls. Differences between controls and patient were tested by t-test using GraphPad Prism 5.0. Values were considered statistically significant with a p-value <0.05. Grubb’s test was used to determine if any of the subjects met the criteria of an outlier.

The use of tissues was approved by the Committee for Ethical Review of Research involving Human Subjects, University of British Columbia. Written informed consent was obtained from the patients’ parents for the publication of this report and any accompanying images.

## Results

### Clinical description

#### Subject 1

**Figure 1:**
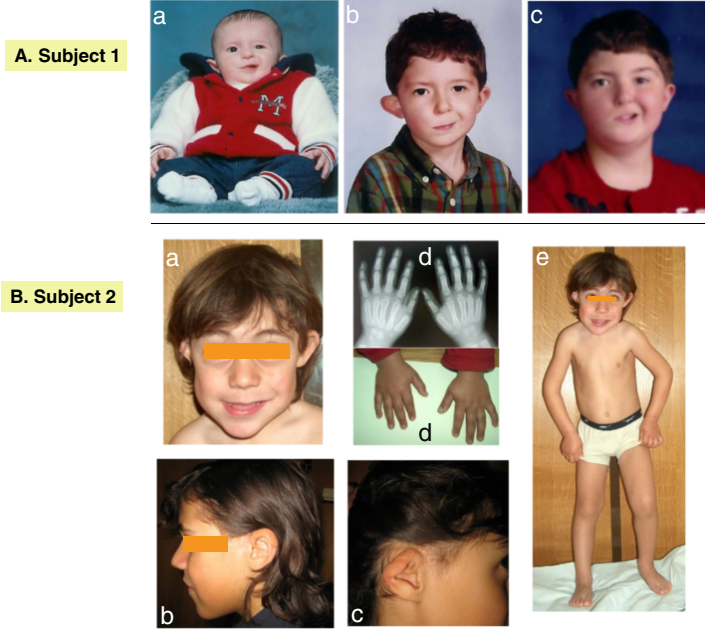
**Clinical presentation of Subject 1 and Subject 2. A**. Subject 1 at age 2, 7, and 13 **(a**-**c)**, demonstrating the facial appearance, with asymmetry, right facial nerve palsy, dysplastic and prominent ears. By age 13, the right ear was surgically corrected. **B**. Subject 2 demonstrating **a)** asymmetry of the jaw, **b)** left ear with thick helix, **c)** right ear (post surgery): small, dysplastic, cup-shaped, anteverted, and hypoplastic lobule, **d)** hands with slightly tapering fingers and **e)** normal body proportions except short neck.

**Figure 2:**
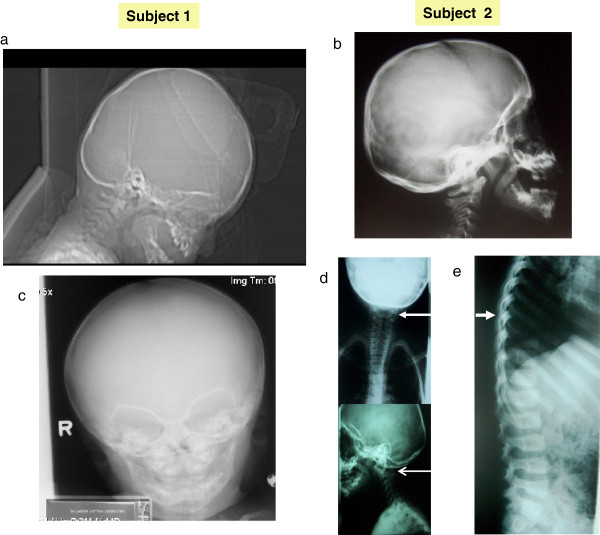
**Skull and skeletal abnormalities in two subjects. a, b)** brachycephaly in Subject 1 **(a)** and 2 **(b)**; **c)** occipital asymmetry in Subject 1; **d)** congenital C1-C2 vertebral block; **e)** accentuation of dorsal kiphosis (arrow) in subject 2.

This male was seen by a geneticist as a neonate because of dysmorphic features, including an asymmetric crying face with a right facial nerve paralysis, a dysplastic right ear, brachycephaly (Figures 1 and 2) and mild contractures of the knees and elbows (which disappeared by five months). The pregnancy was complicated by maternal nausea and vomiting, with only a five pound maternal weight gain. Birth weight was on the 10th centile. A 2 mm secundum atrial septal defect closed in early infancy. His parents are not consanguineous. A paternal great-uncle has intellectual disability while another paternal great-uncle’s adult son has “autistic like features” at age 40. The remainder of the family history is unremarkable.

As an infant, he had difficulty transitioning to solid foods, with gagging and vomiting. He walked at 13 months. At 22 months, he had only a few words. By three years, he was not using any words regularly although he would use a word for a few days and then stop. With most activities, he was not able to stay on track, being quite hyperactive and distractible. His clinical diagnosis of autism was confirmed by an assessment using ADOS at age 2 ½ years. At 13 years of age, his academic functioning was somewhat variable, with mathematics being comparable to the skill level of a ten year old, while the level for other subjects was comparable to an eight year old.

At 22 months, height was above the 50th centile, weight was below the 10th centile, and OFC was on the 3rd centile. He had brachycephaly, which had been seen in infancy. The metopic suture, which was open at 7 months, was closed at 22 months. The left ear was low set, while the right was dysplastic, prominent, with a thin helix and an abnormal crural fold. His face appeared to be asymmetric, due to the right facial nerve palsy. Eye examination was normal except for right eyelid paralysis, secondary to the right facial nerve palsy. The remainder of the physical examination was unremarkable.

Chromosome testing was normal, including FISH at 22q11.2. A CT scan of the brain was normal. Skull radiographs at age 7 months showed a shortened anterior-posterior diameter relative to width. There was slight asymmetry of the appearance of the orbits, with the left side a little larger and slightly more prominent superiorlaterally (Figure 2). No obvious sutural stenosis was evident. At age 14, a C-spine radiograph was normal.

#### Subject 2

The Subject is a 9 year old boy referred for a genetics consultation because of delayed milestones and dysmorphic faces. His parents are non-consanguineous and he has one healthy sister. His maternal uncle died at the age of 3 months, of unexplained sudden death. No other family history of developmental problems or congenital anomalies was reported. At the age of 3 years, he had two episodes that raised the suspicion of absence seizures, but the EEG was normal. Delayed development was noted, especially in language. He spoke few words with no sentences. Mild motor delay also was described, with unaided walking starting at 16 months.

At four years, his growth parameters were normal (weight: 75th percentile; height: 50-75th percentile and OFC: 50-75th percentile). At five years he had a global developmental quotient of 53.4 on the Griffiths Mental Developmental Scale evaluation. His I.Q. at the age of 7 years based on the Wechsler Intelligence Scale was 73 (borderline mental retardation), with a verbal scale of 66 and performance scale of 89. The proband was dysmorphic with a triangular face, brachycephaly, hypertelorism, up-slanting palpebral fissures, thin lips, hypertrophic gums, a pointed chin and short neck (Figure 1). He had abnormal ears (asymmetric, dysplastic and low-set). The right ear was small, cup-shaped, anteverted and the lobule was hypoplastic. The left ear was bigger than the right, with a thick helix. Asymmetry of the jaw was noted, with the left side longer than the right side. His fingers were slightly tapering. Radiographs revealed congenital C1-C2 vertebral fusion, with accentuation of dorsal kyphosis (Figure 2).

Subject 2 displayed stereotypies, aggressive behavior, hyperactivity (including jumping if agitated) and attention deficit. Brain MRI, echocardiogram, abdominal ultrasound, ophthalmology and ENT examinations were normal. Radiographs of upper and lower limbs, pelvis and rib cage did not reveal abnormalities. Chromosome, FISH for the 22q 11.2 region, molecular testing for Fragile X and metabolic studies (plasma aminoacids, urine organic acids, CDT, creatine and guanidinoacetic acid in urine, 7-dehidrocholesterol, lactate, pyruvate and ammonia) were normal.

The clinical description of the two subjects is summarized in Additional file 1: Table S1.

### Array CGH

**Figure 3:**
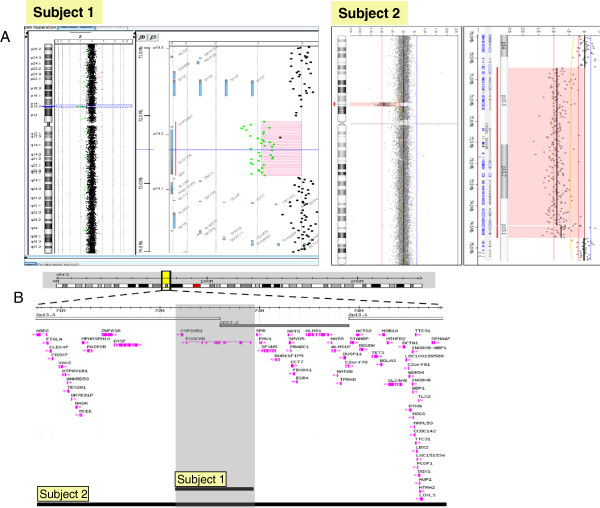
**Array CGH result for Subject 1 and 2. A**. Whole genome array profiles in Subject 1 and 2 showing microdeletions of 2p13; **B**. Gene content for the microdeletions in Subject 1 and 2.

For Subject 1**,** aCGH revealed a 0.78 Mb de novo deletion at chromosome region 2p13.2-13.3 (72,140,702-72,924,626), containing 2 genes. Subject 2 had a 4 Mb *de novo* deletion detected at chromosome region 2p13.1-p13.3 (chr2:70,748,414-74,840,026), containing 62 genes (Figure 3A and 3B). The region of overlap between both cases is 0.78 Mb, located between the 72,140,702-72,924,626 genomic positions and encompasses the *CYP26B1* and *EXOC6B* genes.

### Functional studies of EXOC6B and notch effector genes HES1 and RBPJ in subject 1

**Figure 4:**
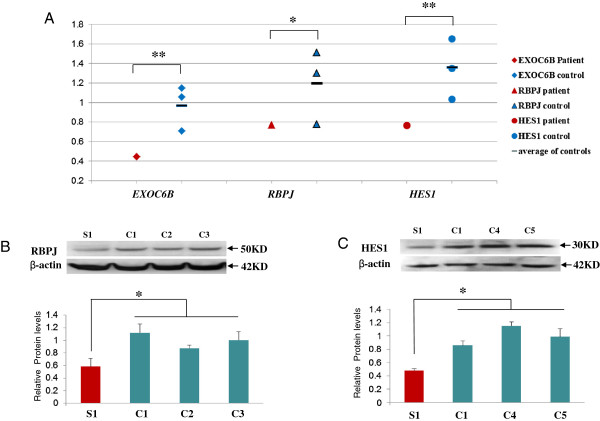
**mRNA and protein expression in Subject 1 and control lymphoblast cells. A**. Scatter graph of mRNA expression of *EXOC6, RPJB* and *HES1*in lymphoblasts. Values for *EXOC6, RPJB* and *HES1* mRNA expression were normalized to the corresponding ß-actin mRNA levels. The results derived from at least three sets of samples and the mean level of each sample is represented in the graph, horizontal bar is an average value between three controls. The difference between the study sample and the average of the controls has been evaluated by student t-test (*, p < 0.05; **, P < 0.01). **B**. RBPJ protein expression. **C**. HES1 protein expression. C1-C5 indicate control samples from the lymphoblasts, S1 indicate study sample from Subject1. Cell lysates were analyzed by SDS-PAGE and immunoblotting with membrane probed for RBPJ, HES1 and ß-actin, Values for RBPJ and HES1 expression were normalized to the corresponding ß-actin levels. The results derived from at least three sets of samples and the mean value of each sample is represented in the bar graph. The difference between Subject1 and the average of three controls has been evaluated by student t-test (*, p < 0.05).

Significant reduction of RNA expression for all three genes (*EXOC6*B, *RBPJ* and *HES1*) was detected in lymphoblasts from Subject 1 in comparison to three controls (Figure 4A). Protein expression for both HES1 and RBPJ was also significantly reduced in his lymphoblasts (Figure 4B and 4C), however, EXOC6B protein level could not be assessed due to a number of non-specific bands or a single band of an unexpected size in controls obtained with three commercial EXOC6 antibodies.

**Figure 5:**
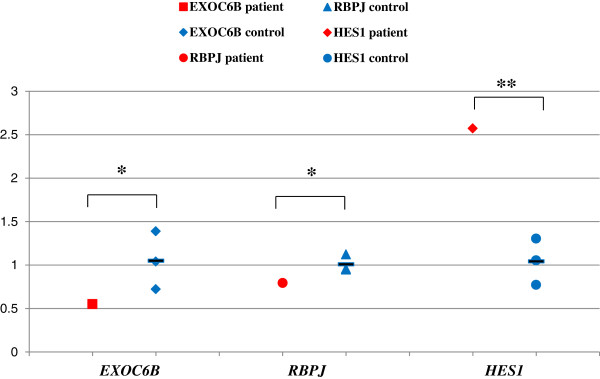
**mRNA expression of *EXOC6, RPJB *and *HES1 *in whole blood of Subject 1 and controls.** Values for *EXOC6, RPJB* and *HES1* mRNA expression in whole blood were normalized to the corresponding ß-actin mRNA levels. The results derived from at least three sets of samples and the mean level of each sample is represented in the scatter graph, horizontal bar is an average value between three controls. The difference between subject 1 and the average of the controls has been evaluated by student t-test (*, p < 0.05; **, P < 0.01).

We noted that in control lymphoblasts, the baseline level of *HES1* and *RBPJ* expression was significantly higher than *EXOC6*, possibly due to the fact that EBV viral protein EBNA2 induces *RBPJ* expression [36] (Figure 4A). To eliminate the effect of EBV transformation on RBPJ and HES expression, whole blood was used from Subject 1 and the controls. Similarly to the lymphoblasts, RNA expression of *EXOC6* and *RBPJ* was significantly reduced in the Subject’s whole blood in comparison to a control. However, *HES1* expression was significantly higher in whole blood of Subject 1 than in the control bloods (Figure 5). Protein from whole blood was not available for assessment of RBPJ and HES1 expression levels.

### ATRA induction of *CYP26B1* expression

**Figure 6:**
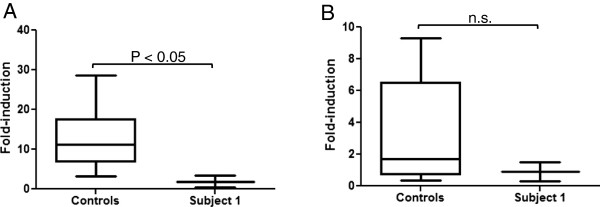
**Induction of *CYP26B1 *and *CYP26A1*. A**. Fold induction of *CYP26B1* mRNA expression. *CYP26B1* was induced significantly more (*P < 0.05) in the control cells as compared to the Subject 1 (S1) cells with a chromosomal deletion of *CYP26B1* after treatment with ATRA. **B**. Induction of *CYP26A1* mRNA expression in ATRA-treated cells. There was no significant difference (n.s.; P > 0.05) in *CYP26A1* induction between the control cells and Subject 1 cells.

EBV transformed lymphoblasts from seven controls and Subject 1 were treated with 1 μM ATRA for 3 days. To obtain a quantitative comparison of the ATRA induction of *CYP26B1* in Subject 1 LCLs versus LCLs from controls, the fold difference in *CYP26B1* mRNA induction with ATRA treatment was calculated for Subject 1 and controls in comparison to the vehicle treated cells. ATRA induction of CYP26B1 mRNA in LCLs from one control subject (56.7 ± 3.7 fold) was determined to be an outlier by Grubb’s test from the other controls and was excluded from further analysis. The *CYP26B1* mRNA fold-induction from the six remaining control cells was averaged to show the range in induction in control LCLs (Figure 6A). *CYP26B1* mRNA induction with ATRA treatment was significantly less in Subject 1 compared to averaged control cells (1.9 ± 1.5 fold in subject and 12.9 ± 7.3 fold in controls,) (Figure 6A).

The relatively minimal expression of *CYP26B1* in ATRA-treated cells from Subject 1 raised the possibility of compensatory expression of *CYP26A1* to regulate retinoic acid levels. ATRA-treatment resulted in minimal induction of *CYP26A1* mRNA in all LCLs which is in agreement with the lack of *CYP26A1* induction and overall low to undetectable expression in blood [18]. *CYP26A1* induction was not significantly different in control LCLs (3.4 ± 3.4 fold) compared to Subject 1 LCLs (0.9 ± 0.9 fold) as shown in Figure 6B. Thus, *CYP26A1* was not induced to a greater extent in response to ATRA treatment in cells with a deletion of *CYP26B1*. To exclude the possibility that *CYP26A1* expression was higher in Subject 1 cells compared to control cells prior to ATRA treatment, we compared the ΔCt values of the vehicle-treated cells and found no differences (P > 0.05). Hence, it does not appear that *CYP26A1* mRNA expression is upregulated to compensate for reduced *CYP26B1* expression in Subject 1 LCLs. While upregulation of *Cyp26a1* has been observed in *Cyp26b1*^-/-^ mice at specific developmental stages and tissues during mouse organogenesis, overall, the expression patterns and function of *Cyp26a1* and *Cyp26b1* are considered non-overlapping during mouse development [37-39].

## Discussion

We report a 2p13.1-p13.3 microdeletion observed in two subjects with clinical effects on the cognitive function (ID and language delay), behaviour (hyperactivity), and development of the craniofacies (facial asymmetry, unusually shaped and asymmetric ears and brachycephaly). Skull and vertebral bone abnormalities included slight asymmetry of the appearance of the orbits and delayed closure of the metopic suture in Subject 1 and congenital C1-C2 vertebral fusion, and accentuation of dorsal kyphosis in Subject 2. Previous cases with cytogenetic deletions/disruptions of this region all had developmental delay and the majority had head/facial anomalies, ear and skeletal abnormalities [1,2].

The overlapping deleted region of our two cases encompasses two genes, *EXOC6B* and *CYP26B1*, both of which showed altered function in whole blood and/or LCLs from Subject 1. The reduced expression of *EXOC6B* in LCLs and whole blood could be the cause of the observed perturbed Notch signaling (i.e. *HES1* and *RBPJ* expression change), considering the similar effect of *EXOC6B* knockout on Notch signaling in *Drosophila.*^20^ The reduction of RNA expression of *EXOC6B* and *RBPJ* was concordant between LCLs and whole blood, and the expression of the *RBPJ* protein also was reduced in Subject 1 lymphoblasts. However, *HES1* had reduced RNA and protein expression in LCLs and increased RNA expression in whole blood. The discrepancy in the pattern of abnormal expression of *HES1* in different cell types could be due to the difference in the Notch signaling pathway in lymphoblasts which contain dedifferentiated B cells vs. whole blood which contains multiple differentiated cell lineages. Cell-specific over or underexpression of *Hes1* has been reported in different regions of the brain in *Rbpj* knockout mice [40].

*EXOC6B* germline mutations or deletions have yet not been reported in humans. A homozygous mutation in *EXOC6B’s* paralogue *EXOC6A* has been reported in mice with hemoglobin-deficit (hbd) due to defective iron transport in the endocytosis cycle [41] while haploinsufficiency of *EXOC6A* due to a 0.3 Mb microdeletion at 10q23.33, was reported in a family with nonsyndromic bi- and unilateral optic nerve aplasia [42]. Interestingly, this microdeletion also included two CYP genes, *CYP26A1* and *CYP26C1*. In contrast to very little information on genetic defects of *EXOC6B* and their role in disease, genetic abnormalities of *HES1* and *RBPJ* have been associated with a number of developmental defects in vertebrates. Homozygous *Hes1* and *Rbpj* knockout mice showed severe developmental defects and lethality [43,44], while *Rbpj* heterozygous knockout mice demonstrated learning deficits [29]. In humans, increased expression of *HES1* was reported in Down syndrome [45] and recent exome sequencing studies revealed heterozygous mutations in *RBPJ* and reduced expression of *HES1* in two families with Adams-Oliver syndrome, associated with congenital cutis aplasia, terminal limb abnormalities (asymmetric shortening of the hands and feet in one of the families) and a range of cognitive functioning (from intellectual disability to normal) [46].

The deletion of *CYP26B1* gene in both our Subjects is also likely to contribute to their abnormal phenotype, based on abnormal RA metabolism in Subject 1, as evidenced by significantly attenuated induction of *CYP26B1* expression with ATRA in comparison to controls. To the best of our knowledge, there are no reports on the effect of *CYP26B1* gene haploinsufficiency in humans. Previously, in two other families, two different homozygous mutations of *CYP26B1* have been reported, resulting in lethality, skeletal and craniofacial abnormalities, including fusion of long bones, calvarial bone hypopasia and craniosynostosis [16]. In one of the families with the hypomorphic mutation, brachycephaly and wide sagittal sutures were noted. The two mutation-bearing constructs had attenuated ability to metabolize ATRA (36% and 86%) [16]. In our Subjects, the decreased RA catabolism and increased sensitivity to RA, as a consequence of *CYP26B1* deletion could explain features similar to those noted by Laue *et al*. [16] (e.g. brachycephaly for both subjects, and for Subject 1, the delayed closure of the metopic suture) and in general compromise the craniofacial, skeletal development and neuronal functioning. With regard to the later phenotype, it is of interest that Subject 1 had an asymmetric crying face as a consequence of a right facial nerve palsy which previously was associated with RA exposure or early embryonic insult [11,47]. The phenotype of the subjects is in agreement with developmental effects of CYP26B1 deletion in experimental animals [37]. CYP26B1 null mice have craniofacial abnormalities, exhibit abnormal ear development and other bone and cartilage deformities [13]. Interestingly, the truncated limbs observed in CYP26B1^-/-^ mice were absent in the patients. Similarly, in the zebrafish CYP26B1 deletion has been shown to result in overall defective craniofacial cartilage development with smaller head, severely decreased number of vagal branchiomotor neurons and defective or absent jaw cartilage [48].

It is intriguing to note the lethal phenotype in two subjects reported by Laue *et al.*[16] due to homozygous mutations of *CYP26B*1 in comparison to the survival and developmental abnormalities in our subjects with hemizygous *CYP26B1* deletion. The presence of one normal copy of the gene in each of our subjects and the efficiencies of the other two remaining RA catabolizing CYP26 genes (*CYP26A1* and *CYP26C1*), possible compensatory changes in other proteins known to regulate retinoic acid, such as RALDH [8,49,50] and environmental influences, such as diet and pharmacological treatment, [6,51] all could have an effect on the phenotypes. Phenotypic variability also was noted for carriers of *CYP26A1, CYP26C1* and *EXOC6A* deletions within one family [42], ranging from normal vision, to uni- and bilateral optic nerve hypoplasia and variable levels of cognitive functioning (normal to impaired).

The combined effect of deletion of both *EXOC6B* and *CYP26B1* on Notch and RA signaling, and consequently the phenotype, also should be considered in our subjects. Interaction of RA and Notch signaling in determination of left/right asymmetry and segmentation has been reported by Echeverri and Oates [52] who noted the requirement of *Rbpj* function for expression of RA catabolizing enzyme *Cyp26a1* which in turn, is needed for left/right symmetric cyclic gene expression. Vermot *et al.*[53] demonstrated that reduced levels of RA were associated with abnormal *Hes1* expression and asymmetry in mouse embryos, while Castella *et al*. [54] showed that addition of RA raises the level of *HES1* protein expression in *in vitro* cell culture.

Subject 2 had additional phenotypic features not noted in Subject 1, which might be explained by the larger size of the 2p13.1-13.3 deletion, interaction of its integral genes and genetic background. For example, seizure-like episodes were not noted in Subject 1, but were present in Subject 2 and a Decipher subject, # 257412, with developmental delay, whose deletion of 2p12-13.3 was 6.8 Mb (70,889,254-77,746,500), and his features included coarse faces, a flat malar region, myopathic hypotonia and prominent ears. The cervical fusion and spine abnormality are unique for Subject 2 and are of interest, considering the report of a subject with Klippel-Fiel anomaly, who had a balanced inversion involving chromosome 2p13 and congenital fusion of the cervical spine, impairment of hearing, psychomotor retardation, speech limitation, short stature, spinal asymmetry and scoliosis [3]. The genes disrupted/deleted by this chromosome rearrangement are unknown, however, it was speculated that *CYP26B1* was involved based on the similar vertebral phenotype observed in zebrafish with *Cyp26b1* mutations [4].

Our report is unique as it provides a new insight into the phenotypic and functional consequences of hemizygous deletion of two genes implicated in Notch and Retinoic acid signaling. It also supports the previous cell line and animal model based observation of exocyst role in Notch signaling. Further studies of exocyst complex function in patient cells would be of interest for understanding of its role in human disease.

## Competing interests

The authors have no competing interests to declare.

## Authors’ contribution

JW, ERS, CN, NI, FL and PM: Conception, design, analysis and interpretation of data. Drafting the article or revising it critically for important intellectual content. SAF, GS, SL, CB, YQ, BY and SM: Analysis and interpretation of data. Drafting the article or revising it critically for important intellectual content. All authors read and approved the final manuscript.

## Authors’ information

Patricia Maciel and Evica Rajcan-Separovic are co-corresponding authors.

## Supplementary Material

Additional file 1: Table S1 Clinical features of Subject 1 and 2. Click here for file
